# Monoterpenes Released from Fruit, Plant, and Vegetable Systems

**DOI:** 10.3390/s141018286

**Published:** 2014-09-29

**Authors:** Mohammad Asif Iqbal, Ki-Hyun Kim, Jeong Hyeon Ahn

**Affiliations:** Atmospheric Environment Laboratory, Department of Civil and Environmental Engineering, Hanyang University, 222 Wangsimni-ro, Seoul 133-791, Korea; E-Mails: iqbaldu88@gmail.com (M.A.I.); qqq112311@gmail.com (J.H.A.)

**Keywords:** monoterpene, thermal desorption, impinger, 3-bed sorbent tube, emission

## Abstract

To quantify the emission rate of monoterpenes (MTs) from diverse natural sources, the sorbent tube (ST)-thermal desorption (TD) method was employed to conduct the collection and subsequent detection of MTs by gas chromatography. The calibration of MTs, when made by both mass spectrometric (MS) and flame ionization detector (FID), consistently exhibited high coefficient of determination values (R^2^ > 0.99). This approach was employed to measure their emission rate from different fruit/plant/vegetable (F/P/V) samples with the aid of an impinger-based dynamic headspace sampling system. The results obtained from 10 samples (consisting of carrot, pine needle (*P. sylvestris*), tangerine, tangerine peel, strawberry, sepals of strawberry, plum, apple, apple peel, and orange juice) marked α-pinene, β-pinene, myrcene, α-terpinene, *R*-limonene, γ-terpinene, and *p*-cymene as the most common MTs. *R*-limonene was the major species emitted from citrus fruits and beverages with its abundance exceeding 90%. In contrast, α-pinene was the most abundant MT (37%) for carrot, while it was myrcene (31%) for pine needle. The overall results for F/P/V samples confirmed α-pinene, β-pinene, myrcene, α-terpinene, and γ-terpinene as common MTs. Nonetheless, the types and magnitude of MTs released from fruits were distinguished from those of vegetables and plants.

## 1. Introduction

The environmental significance of biogenic volatile organic compounds (BVOCs) is well known for their potent role in the formation of the tropospheric ozone like their anthropogenic counterparts [1–3]. As the major components of BVOC, monoterpenes (MTs: C_10_H_16_) are formed as the secondary metabolites of plants with two isoprene units (C_5_H_8_). They are also the key components of the fragrant or essential oils obtained from vegetables (e.g., carrot), plant segments (e.g., pine needle), and fruits [4–6]. The flavor of highly complex beverages (e.g., wines and juices) is also dominated by essential fragrance components like MTs [6–8]. A recent review focusing on the cardiovascular effects of MTs marked their promising role in the prevention or treatment of such diseases [9].

In light of the potent role of MTs in atmospheric chemistry, researchers have had a great deal of interest in their quantification as tracers of organic aerosols [10]. In laboratory-based quantitation of MTs, gas chromatography (GC) equipped with mass spectrometric (MS) or flame ionization detector (FID) is a common choice [5,11,12]. Instrumental setups like multidimensional gas chromatography–mass spectrometry (MD/GC/MS) were also applied in some previous studies [13,14]. Some analytical techniques developed and introduced recently (such as selected-ion-flow-tube mass spectrometry (SIFT-MS) and Proton-transfer-reaction mass spectrometry (PTR-MS)) greatly enhanced the level of sensitivities in the detection of VOCs including many MTs [11,15]. In the analysis of MTs, a critical component is the selection of proper pretreatment methods, with such options as solid phase extraction (SPE: [14]), solid phase microextraction (SPME: [16]), and TD-based analysis with multiple-bed sorbent tubes (STs) [12].

This study was carried out to offer insights into the accurate quantification of MTs released from fruit/plant/vegetable (F/P/V) systems through the application of both the FID and MS detectors. To this end, the feasibility of the ST/TD method was explored through dynamic headspace analysis. The results of this study will highlight the basic characteristics of MT emissions from diverse F/P/V samples. Based on this study, we will also discuss relative dominance of MTs between different F/P/V samples.

## 2. Materials and Method

In this study, a quartz tube packed with three different layers of sorbent materials (Tenax TA, Carbopack B, and Carbopack X (namely TBX)) was used for the collection of MTs from the real samples selected in this study. Calibration of MTs was also done through the absorption of liquid-phase standards on these STs for subsequent analysis by both TD/GC/MS and TD/GC/FID systems. For the actual measurements of MTs from a total of 10 F/P/V samples (e.g., carrot, pine needle (*P. sylvestris*), tangerine, tangerine peel, strawberry, sepals of strawberry, plum, apple, apple peel, and orange juice), dynamic headspace sampling was carried out by a small flux chamber system [17,18].

### 2.1. Preparation of Standards and Calibration

As listed in Table 1, a total of 9 MTs and 1 alkylbenzene related to MT (*p*-cymene) were selected as the target compounds: α-pinene (1), camphene (2), β-pinene (3), 3-carene (4), myrcene (5), α-phellandrene (6), α-terpinene (7), *R*-limonene (8), γ-terpinene (9), and *p*-cymene (10). In addition, to check the system performance, toluene was added as a reference compound. Liquid-phase working standards (L-WS) containing all MTs were prepared at six different concentration levels (5, 10, 20, 50, 100, and 300 ng/µL) by three step dilutions of reagent grade chemicals (purity: 95.0%∼99.5%; purchased from Sigma–Aldrich, St. Louis, MO, USA) (Table S1). After preparation, L-WSs were stored in six 1.5 mL size vials (Agilent Technologies, Santa Clara, CA, USA).

**Figure 1.**
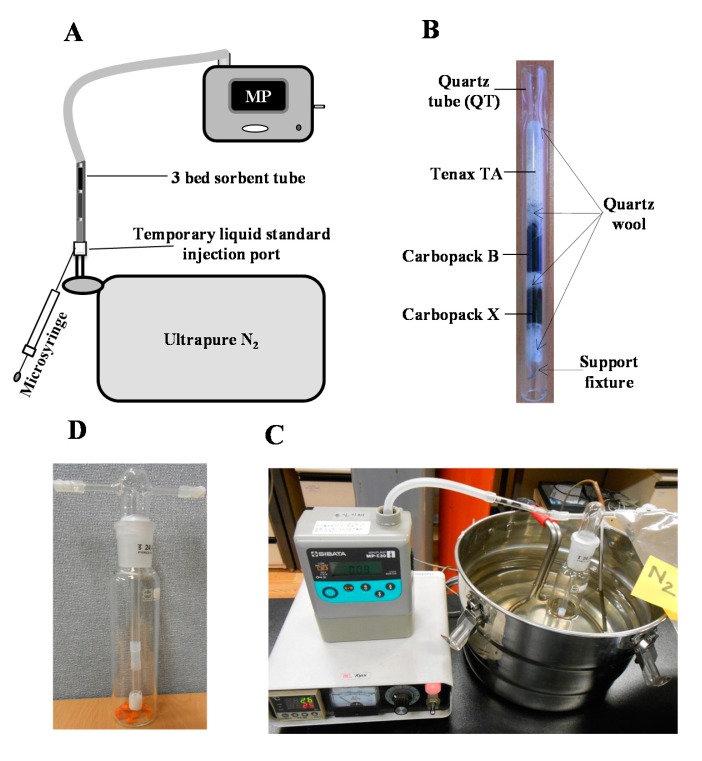
Schematic diagrams of monoterpenes (MTs) sampling apparatus and setup: (**A**) diagram of loading L-WS on ST; (**B**) picture of ST with 3-bed components; (**C**) experimental setup for headspace sampling, and (**D**) impinger containing sliced carrot samples.

To conduct the calibration of MTs, 1 µL of L-WS was directly loaded (via syringe) on the 3-bed ST described above (Figure 1A,B). In this system, the inlet and outlet of the ST were respectively connected to a 10 L polyester aluminum (PEA) bag filled with ultra-pure N_2_ (99.999%: as back-up gas) and a vacuum pump interfaced with a mass flow controller (Sibata ΣMP-30, Japan) (Figure 1A). L-WS was directly injected onto the ST via a temporary injection port made by Teflon tube that connected the inlet of the ST and the polyester aluminum (PEA) bag; the back-up gas was supplied from the PEA bag to the ST at a constant flow rate of 100 mL min^−1^ for 5 min. STs loaded with L-WS of MTs were placed on TD for thermal desorption analysis. For the application of this procedure, five point calibrations of MTs were done independently by TD/GC/MS and TD/GC/FID systems.

### 2.2. Impinger System for the Emission Rate Measurements for F/P/V Samples

In this research, volatile aroma compounds and MTs were measured from diverse F/P/V samples. To measure their emission rates, an impinger-based headspace collection system was employed (Figure 1C,D), as reported in our previous study [17,18]. To perform headspace sampling of MTs, fresh F/P/V samples were purchased from a nearby market (Gunja, Seoul, Korea) or collected from the campus grounds of Sejong University. For fruit samples, edible parts (e.g., tangerine, strawberry, and apple) and commonly non-edible parts (e.g., tangerine peel, apple peel, and sepals of strawberry) were separated for the acquisition of the respective data sets. Samples like carrot, tangerine peel, strawberry, plum, apple, and apple peel were then sliced into small cube shapes (size of each piece approximately 0.075–0.09 cm^3^) using knife, while sample such as pine needle, tangerine, and strawberry were kept in original shape. After that, 1 g of sample was placed in an impinger (175 mL capacity, Schott Duran, Main, Germany) of which the temperature was maintained at 25 °C (Figure 1D). The impinger was then sealed to prevent any leakage of MTs. The collection of headspace sample was made subsequently by supplying ultra-pure N_2_ (99.999%) as sweep gas at a flow rate of 100 mL min^−1^ for 2.5 min (total sampling volume 0.25 L). In the case of orange juice, a 1 mL of sample was placed in the impinger using micropipette (Life Tech., Warsaw, Poland). Then, the headspace sample was collected via the same procedures described above. In addition, ∼1 g of pine needle collected freshly from a pine tree (*P. sylvestris*) was also placed in an impinger (without slicing) for the collection of headspace gas. Once the headspace sample was collected on ST, it was detached from impinger and placed on the TD system for GC/MS analysis.

### 2.3. Instrumental Setup and Operational Conditions

In this study, multiple-bed STs were employed for the simultaneous quantitation of a wide range of MTs. In many previous studies the use of multiple-bed STs has been made as reliable choice for the simultaneous quantification of a wide range of the biogenic volatile organic compounds (BVOCs: e.g., MTs, isoprene, sesquiterpenes, and diterpenes) [12]. Table S2 presents the basic information regarding STs and the instrumental setup for ST/TD/GC analysis. To induce adsorption of MTs, STs were prepared in quartz tube packed with three different layers of sorbent materials: weaker sorbent-Tenax TA (60/80 mesh, Restek, Bellefonte, PA, USA), medium sorbent-Carbopack B (60/80 mesh, Supelco, St. Louis, MO, USA), and strong sorbent-Carbopack X (40/60 mesh, Supelco, St. Louis, MO, USA); 50 mg of each were placed in order in a quartz tube to allow quantitative recovery of target MTs in consideration of their chemical characteristics (e.g., vapor pressure) (Figure 1B). Generally, the low *vapor pressure* compounds (e.g., MTs) are retained on the weaker sorbent (e.g., Tenax TA). In the TD unit, the tube was back-flushed to keep the lower *vapor pressure* compounds unexposed to the stronger sorbent [12].

For GC/FID analysis, GC (Varian GC; Agilent Technologies, USA) equipped with a multifunction TD (UNITY, Markers International Ltd., UK) was used. For GC/MS analysis, Shimadzu GC-MS was equipped with another TD with the same configuration. In both GC/MS and GC/FID analysis, polar column (CP-WAX; Varian, Santa Clara, CA, USA) was used for chromatographic separation of MTs collected from ST samples. The details of temperature programming for the operation of both GC/MS and GC/FID systems are provided in Table S2.

### 2.4. Basic Quality Assurance of TD-GC-System between the Two Detectors

To assess the relative performance between MS and FID in the application of ST/TD/GC, the basic quality assurance parameters were evaluated with respect to the method detection limit (MDL) and reproducibility (via relative standard error: RSE (%)). These quality assurance parameters were determined by seven and three replicate analyses of the lowest calibration point L-WS (about 5 ng of each compound), respectively (Table 3S). The MDL values were calculated as the product of SD and the student’s t-value (3.14) at a 99% confidence level [20].

In the MS system, the MDL values were found from 0.23 (camphene) to 0.50 ng (*R*-limonene). These MDL values, if expressed in terms of concentration such as nmole/mole (or ppb) unit by assuming the sample volume of 0.5 L, fell in the range of 0.08 (camphene) to 0.18 ppb (*R*-limonene). In FID, the MDL values were found in the range of 0.38 (0.14 ppb for α-pinene) to 0.89 ng (0.32 ppb for camphene). If the performance of the two detectors (FID *vs.* MSD) is compared by means of average mass-based MDL values of MTs, the latter revealed approximately two-fold enhanced sensitivity relative to the former. The precision of MT analysis using the MS detector was in the range of 0.50 (α-pinene) to 3.76% (β-pinene), while that of FID was 0.50 (camphene) to 4.04% (3-carene). If averaged RSE (%) values are compared, the MS results were slightly better (0.32%) than FID (Table S3).

## 3. Results and Discussion

### 3.1. Basic Detection Properties of MTs between FID and MS

**Figure 2.**
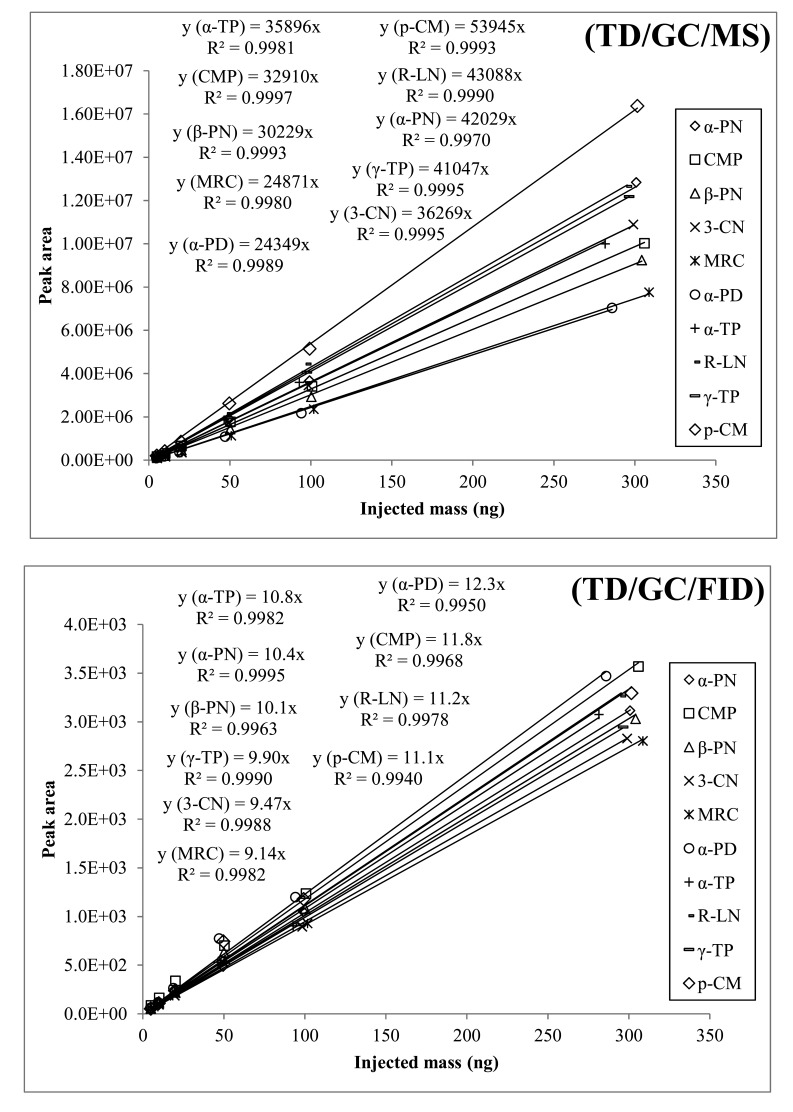
Calibration results of all target MTs selected in this study for both TD-GC-FID and TD-GC-MS analyses.

**Figure 3.**
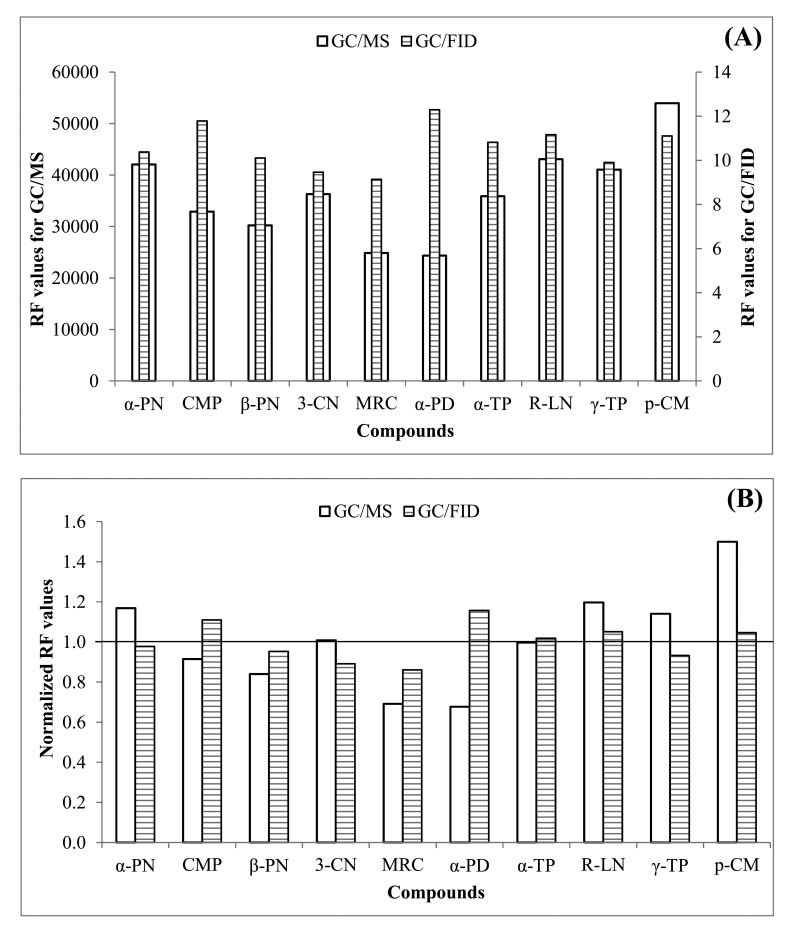
Comparison of both (**A**) absolute and (**B**) normalized response factor (RF) values of all target MTs between GC/MS and GC/FID.

In this study, the system performance of both the GC/MS and GC/FID methods was examined in the analysis of MTs. However, quantification of real samples was made only by GC/MS. To complete six point calibrations of MTs, L-WS was directly injected in ST with the aid of microsyringe with the supply of ultra-pure N_2_ (Figure 1A). The calibration results of both FID and MS generally yielded the coefficient of determination (R^2^) at around >0.99 (Figure 2). In case of FID, the response factor (RF) values were in a very close range (9.14 (myrcene)-12.3 (α-phellandrene)). In contrast, those of MS exhibited two fold variations (24349 (α-phellandrene)-53945 (*p*-cymene)). The highest variation between MS and FID RF was observed in case of α-phellandrene; it gave the highest response (among all MTs) in FID detector (12.3), while being the lowest (among all MTs) in MS analysis (24349) (Figure 3A). The normalized RF values of MTs obtained by both systems are also compared in Figure 3B. The normalized RF values for each compound were obtained for a particular detector (e.g., MS) by dividing the RF values of individual MTs with their mean for all MTs. Comparison of these normalized RF values (between FID and MS) exhibited minimal difference for most MTs (except α-phellandrene and *p*-cymene).

**Figure 4.**
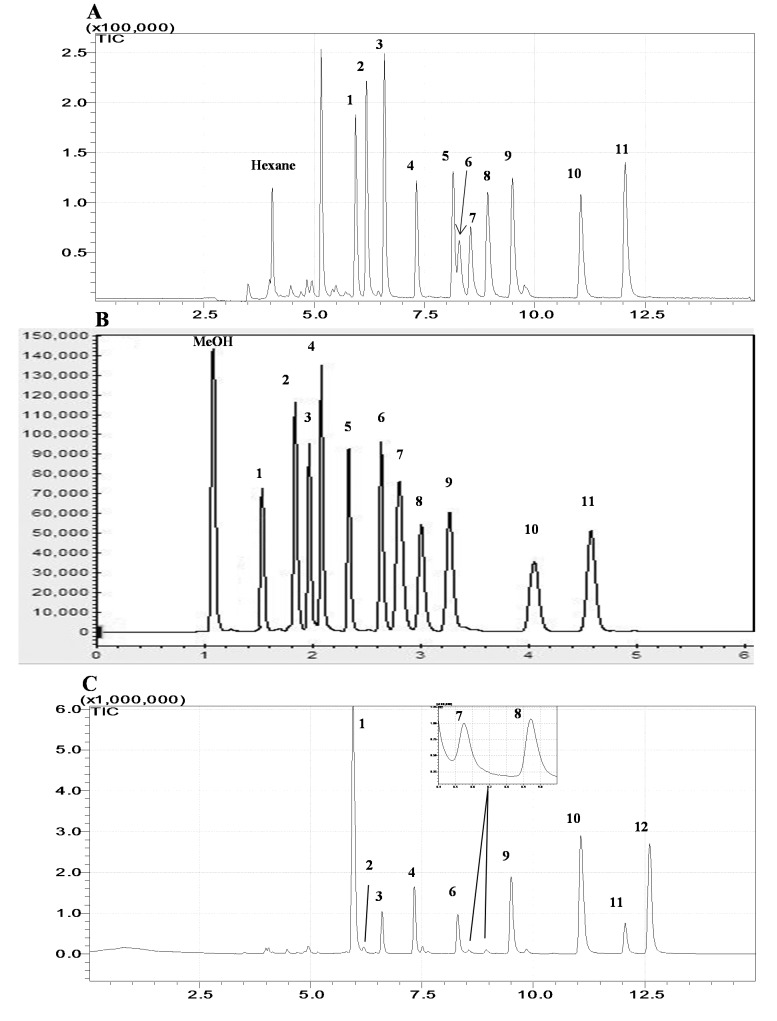
Representative chromatograms of MTs obtained by ST/TD/GC system: (**A**) L-WS of 10 ng (MS); (**B**) L-WS of 50 ng (FID); and (**C**) 250 mL headspace sample of carrot (MS) with the peak ID: α-pinene (1), toluene (2), camphene (3), β-pinene (4), 3-carene (5), myrcene (6), α-phellandrene (7), α-terpinene (8), *p*-cymene (9), *R*-limonene (10), γ-terpinene (11), and α-Terpinolene (12).

In Figure 4, the chromatograms of MTs obtained by both (A) GC/MS and (B) GC/FID are presented. For both detectors, the elution order of investigated MTs were seen on the order of α-pinene, toluene, camphene, myrcene, β-pinene, α-phellandrene, 3-carene, α-terpinene, *p*-cymene, *R*-limonene, and γ-terpinene; this relative ordering complied well with those of the retention index values for these MTs with polar column such as 1040, 1045, 1066, 1118, 1145, 1174, 1176, 1177, 1203, 1244, and 1280, respectively [19]. In GC/MS analysis, several MTs (e.g., 3-carene, myrcene, and α-phellandrene) eluted in a close range (5th, 6th, and 7th peaks, respectively in Figure 4A) in compliance with their very close Kovats retention index values (Table 1) [21].

### 3.2. The Results of F/P/V Sample Analysis

In this study, the emission rates of MTs were measured from diverse samples including (1) carrot, (2) pine needle, (3) tangerine, (4) tangerine peel, (5) strawberry, (6) sepals of strawberry, (7) plum, (8) apple, (9) apple peel, and (10) orange juice (Table 2). For the measurements of BVOC emission rates from these samples, an impinger-based chamber system was employed to collect 0.25 L headspace samples (using multiple-bed STs) for each target (Figure 1).

Table 2 presents the results of our MT analysis from diverse F/P/V samples in two different units/fashions: (A) headspace concentration of MTs (ppm) and (B) emission flux per mass [µg of MT/g of F/P/V samples]. Among all target MTs detected in headspace samples of carrot, the concentration α-pinene was seen as the highest (0.51 ppm). In the analysis of headspace collected from pine needle, α-pinene, myrcene, and *R*-limonene were dominant, while almost all other target MTs were also detected above DL. The headspace concentration of *R*-limonene was very high in all different fruit samples: tangerine (0.60 ppm), tangerine peel (80 ppm), strawberry (0.40 ppm), sepals of strawberry (0.58 ppm), and orange juice (3.22 ppm). The emission flux (µg/g) of MTs also varied widely among different F/P/V samples; their values maintained the relative ordering as follows: tangerine peel > orange juice > pine needle > carrot > tangerine> sepals of strawberry > strawberry (Table 2B). In the case of carrot, the emission flux was mostly dominated by α-pinene, β-pinene, myrcene, *R*-limonene, and γ-terpinene. An emission pattern similar to carrot was also observed from pine needle with notable emission of α-terpinene. The highest emission flux (µg/g) of MTs was observed in the headspace analysis of tangerine peel (12.2 µg/g) and orange juice (4.66 µg/g), which was dominated by *R*-limonene.

**Figure 5.**
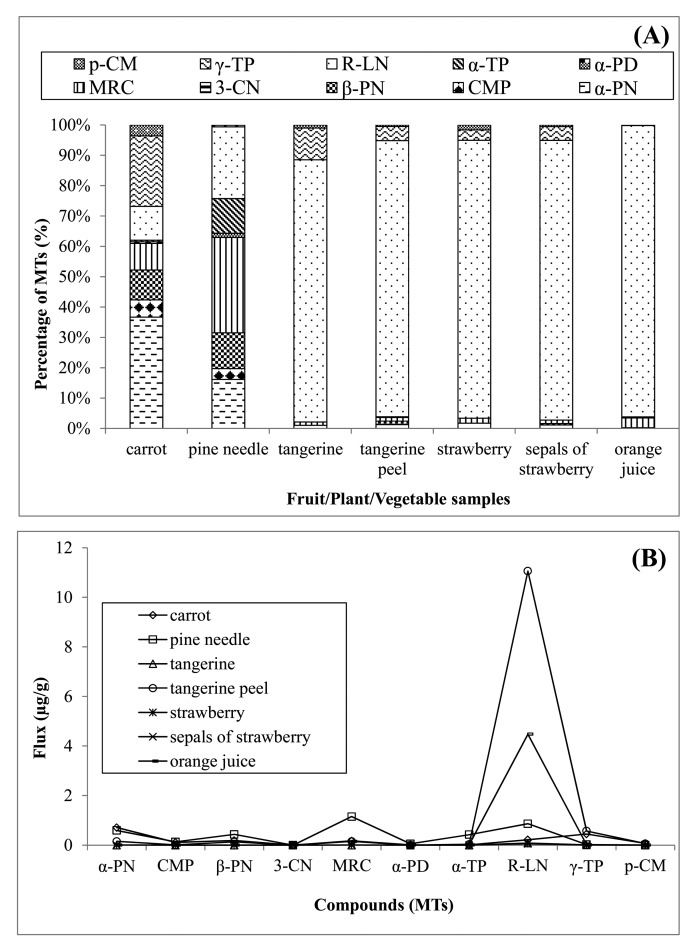
Emission of MTs from fruit/plant/vegetable (F/P/V) samples: (**A**) Relative composition (%) of MTs in collected headspace samples and (**B**) Emission (µg/g) patterns of MTs between different F/P/V samples.

As presented in Figure 5A, *R*-limonene was also the major MT component of strawberry and sepals of strawberry. In the case of tangerine peel, the contribution of *R*-limonene was as high as 91% in total MT-flux. It was also the major contributor to the total MT-flux from tangerine (91%), strawberry (92%), sepals of strawberry (92%), and orange juice (96%). On the other hand, the relative contribution of *R*-limonene in carrot and pine needle was below 25% (11% and 24%, respectively). In the case of carrot, α-pinene was the most abundant MT (37%), while it was myrcene (31%) in pine needle. However, the overall results indicate that some compounds (like α-pinene, β-pinene, myrcene, α-terpinene, and γ-terpinene) are the most common MTs released from vegetable/plant samples, unlike fruit samples.

Figure 5B presents the MT-emission patterns between all different F/P/V samples. In the case of two plant/vegetable samples (carrot and pine needle), the relative dominance of α-pinene, β-pinene, myrcene, α-terpinene, and γ-terpinene were observed. *R*-limonene was the most abundant MT which was seen in the following order from different samples: tangerine peel > orange juice > pine needle > carrot > tangerine > sepals of strawberry > strawberry. It was also interesting to notice that the emission flux (µg/g) of *R*-limonene was high from non-edible parts (tangerine peel and sepals of strawberry) of fruits relative to commonly edible portions (e.g., tangerine and strawberry).

### 3.3. Comparison of Our F/P/V Sample Analysis Data with Previous Studies

In order to evaluate the results of the F/P/V analysis, we also compared with our results with those reported previously (Table 3). Based on the static headspace analysis (SHA), [22] measured α-pinene, camphene, β-pinene, myrcene, α-terpinene, *R*-limonene, and γ-terpinene from seven types of fresh carrots. Although headspace concentrations (µg/L of headspace) of MTs varied, γ-terpinene was observed as the most dominant component [22]. [23] also conducted a headspace analysis of blended carrots (6 types) and reported an emission rate of MTs (µg/g of carrot) in the range of 0.01 (γ-terpinene: Danvers-2) to 3.02 µg/g (α-pinene: Gold Pak). The emission flux of MTs (µg/g/h) were also measured from four types of carrots to show the release of α-pinene, camphene, β-pinene, myrcene, α-terpinene, *R*-limonene, and γ-terpinene [21]. The overall results of these studies marked α-pinene, camphene, β-pinene, myrcene, *R*-limonene, α-terpinene and γ-terpinene as the major MTs emitted from carrots. As such, our observations on carrot samples are highly comparable to the results of those previous works on carrots [21–23] (Table 3A).

According to [24], significant amounts of MTs were reported to be released from fresh to decomposed Scots pine needles. These authors used the relative composition of MTs to explain the pine needle decomposition process, as many MTs tend to decrease/disappear with the increase in decomposition time (Table 3B). Another study also reported the emission flux (µg/m^2^/h) of two different MT species (α-pinene and β-pinene) from various grasses (e.g., mixed grasses, Bermuda grasses, Pensacola grasses, and Saw grasses) [25]. *Pelargonium hortorum* leaves were also reported to release α-pinene, camphene, β-pinene, myrcene, and *R*-limonene [26]. The results of those previous studies marked α-pinene, camphene, β-pinene, myrcene, and *R*-limonene as the most common MTs emitted from different plant systems [24–27]. From this point of view, our results are fairly compatible with others, as all different MTs (except 3-carene) were seen in our analysis of pine needle (Table 3B).

In our study, *R*-limonene is marked as the most common MT from fruits and a fruit-derived beverage (orange juice) (Table 3C). For instance, the headspace concentration and emission rate of *R*-limonene was measured as 17.9 µg/L and 4.47 µg/mL, respectively. A previous study made by the SPME analysis of orange juice [28] reported a very high (239 µg/L) HS concentration of *R*-limonene. Another study also reported a significant release of *R*-limonene from orange wine, although the observed emission rate was low (0.43 µg/mL of orange wine) compared to our study (4.47 µg/mL of orange juice) [29].

## 4. Conclusions

In this study, an ST/TD/GC/MS-based analytical technique was developed for the analysis of MTs emitted from diverse F/P/V samples. At the initial stage, the calibrations and basic quality assurance experiments of MTs were done using both an MS and FID detector. The sensitivity of the MS detector was almost two times higher than that of FID. The chromatographic separation in WAX column was also proper, while there was a very good matching of the retention order with Kovats RI values for all target MTs. In the next stage, an impinger-based chamber system was employed to collect MTs (in multiple-bed STs) from F/P/V samples using the dynamic headspace sampling technique. The highest emission was measured from the peel of tangerine, followed by orange juice, pine needle, and carrot. The relative composition of MTs detected from headspaces was distinguished between samples types, as the emission pattern of MTs varied across different F/P/V samples. In the case of vegetable and plant samples, the domination of some MTs such as α-pinene, β-pinene, myrcene, α-terpinene, and γ-terpinene was consistent, while *R*-limonene was the single predominant component in fruit samples with more than 90% abundance in all cases. A comparison of our results shows agreement with available literature data. The results of our study thus indicate that the method developed in this study can be easily employed for rapid and effective measurements of volatile flavor components from diverse vegetable, plant, fruit, and beverage samples.

## Supplementary Material



## Figures and Tables

**Table 1.** Basic information of 10 monoterpenes (MTs) and a reference compound investigated in this study.

**Order**	**Group**	**Full Name**	**Short Name**	**Formula**	**MW (g/mol)**	**Density (g/cm^3^)**	**Kovats RI ^b^**
1		α-pinene	α-PN	C_10_H_16_	136.23	0.858	1045
2		Camphene	CMP	C_10_H_16_	136.23	0.866	1066
3		β-pinene	β-PN	C_10_H_16_	136.23	0.872	1118
4		3-carene	3-CN	C_10_H_16_	136.23	0.857	1145
5	Monoterpenes	Myrcene	MRC	C_10_H_16_	136.23	0.791	1174
6	(MTs)	α-phellandrene	α-PD	C_10_H_16_	136.23	0.850	1176
7		α-terpinene	α-TP	C_10_H_16_	136.23	0.837	1177
8		*R*-limonene	*R*-LN	C_10_H_16_	136.23	0.842	1203
9		γ-terpinene	γ-TP	C_10_H_16_	136.23	0.850	1244
10	Alkylbenzene	*p*-cymene	*p*-CM	C_10_H_14_	134.21	0.860	1280

11	Reference ^a^	Toluene	T	C_7_H_8_	92.14	0.870	1040

a Aromatic volatile organic compound;

b Kovats RI values of MTs for polar column (polyethylene glycol as stationary phase) [19].

**Table 2.** Result of MT emission measurements based on headspace analysis of fruit/plant/vegetable samples using ST/TD/GC/MS system.

**Order**	**Sample Name ^a^**	**α-PN**	**CMP**	**β-PN**	**3-CN**	**MRC**	**α-PD**	**α-TP**	***R-LN***	**γ-TP**	***p*-CM**	

**A. Emission Concentration (ppm) ^b^**												**Total Concentration (ppm)**
1	Carrot ^c^	0.512	0.080	0.139	0.0004 ^e^	0.122	0.008	0.008	0.155	0.323	0.051	1.40
2	Pine needle ^c^	0.424	0.092	0.309	0.0004	0.826	0.035	0.305	0.620	0.011	0.003	2.62
3	Tangerine ^d^	0.006	0.0002	0.0004	0.0004	0.006	0.0002	0.0004	0.595	0.040	0.009	0.66
4	Tangerine peel ^d^	1.081	0.041	0.966	0.0004	1.093	0.0002	0.204	79.55	4.073	0.408	87.4
5	Strawberry ^d^	0.008	0.0002	0.000	0.0004	0.004	0.0002	0.0004	0.389	0.017	0.006	0.42
6	Sepals of strawberry ^d^	0.008	0.0002	0.003	0.0004	0.005	0.0002	0.0004	0.581	0.030	0.003	0.63
7	Orange juice ^c^	0.003	0.001	0.0004	0.003	0.111	0.003	0.006	3.219	0.007	0.0003	3.35

**B. Emission Flux (µg/g of Sample Placed on Impinger) ^f^**												**Total Flux (µg/g)**

1	Carrot ^c^	0.711	0.111	0.193	0.0004	0.170	0.011	0.011	0.216	0.449	0.070	1.94
2	Pine needle ^c^	0.589	0.128	0.429	0.0004	1.148	0.049	0.424	0.862	0.016	0.004	3.65
3	Tangerine ^d^	0.001	0.0002	0.0004	0.0004	0.001	0.0002	0.0004	0.083	0.006	0.001	0.09
4	Tangerine peel ^d^	0.150	0.006	0.134	0.0004	0.152	0.0002	0.028	11.06	0.566	0.056	12.2
5	Strawberry ^d^	0.001	0.0002	0.0004	0.0004	0.001	0.0002	0.0004	0.054	0.002	0.001	0.06
6	Sepals of strawberry ^d^	0.001	0.0002	0.000	0.0004	0.001	0.0002	0.0004	0.081	0.004	0.000	0.09
7	Orange juice ^c^	0.004	0.001	0.0004	0.005	0.155	0.005	0.009	4.474	0.010	0.0003	4.66

a Fruit samples like plum and apple was not added in Table as no MT was detected in headspace collected from those samples;

b Concentration of MTs in headspace (0.25 L) collected from F/P/V samples;

c Sample mass placed on impinger: 1 and 10 g, respectively;

d Sample mass placed on impinger: 1 and 10 g, respectively;

e Not detected: MDL values are given at “µg” unit;

f Emission rate = [Total mass of MTs in 0.25 L headspace / Sample (F/P/V) mass placed on impinger].

**Table 3.** Comparison of MT emission data between different studies.

**Order**	**Sample Type**	**Sampling and Pretreatment^a^**	**Detector (GC)**	**Unit**	**Compounds**										**Reference**

					**α-PN**	**CMP**	**β-PN**	**3-CN**	**MRC**	**α-PD**	**α-TP**	**R-LN**	**γ-TP**	**p-CM**	**No ^b^**

**A. Vegetable (Carrot)**															
1	Carrot (*D. carota*) (CA) ^c^	ST/TD	MS	µg/L of HS	2.85	0.44	0.77	- ^d^	0.68	0.04	0.05	0.86	1.80	0.28	This study
				µg/g	0.71	0.11	0.19	-	0.17	0.01	0.01	0.22	0.45	0.07	
				µg/g/h ^e^	17.1	2.66	4.64	-	4.08	0.26	0.27	5.17	10.8	1.69	
2	Carrot (7 varieties)	SHA	MS	µg/L of HS	0.6–11	0.01–0.08	0.2–1.2		0.6–98	0.2–0.8	0.02–0.2	0.6–9.0	1.1–10	0.03–0.7	1
3	Carrot (Imperator-1)	HS/DI	FID	µg/g	0.22	-	0.43	-	0.66	-	-	-	0.35	-	2
4	Carrot (Imperator-2)	HS/DI	FID	µg/g	0.66	-	0.84	-	0.97	-	-	1.22	1.07	-	2
5	Carrot (Gold Pak)	HS/DI	FID	µg/g	3.02	-	0.16	-	0.36	-	-	0.41	0.65	-	2
6	Carrot (Danvers-1)	HS/DI	FID	µg/g	0.08	-	0.05	-	0.05	-	-	0.07	0.11	-	2
7	Carrot (Danvers-2)	HS/DI	FID	µg/g	0.16	-	0.05	-	0.07	-	-	0.11	0.01	-	2
8	Carrot (Nantes)	HS/DI	FID	µg/g	0.80	-	0.11	-	0.05	-	-	0.03	0.09	-	2
9	Carrot (Brasilia)	HS/LVI	MS	µg/g/h	0.06	0.003	0.01	-	0.04	-	0.002	0.03	0.12	0.11	3
10	Carrot (Duke)	HS/LVI	MS	µg/g/h	0.10	0.003	0.02	-	0.03	-	0.002	0.02	0.06	0.07	3
11	Carrot (Fancy)	HS/LVI	MS	µg/g/h	0.18	0.01	0.04	-	0.17	-	0.003	0.04	0.17	0.23	3
12	Carrot (Cortez)	HS/LVI	MS	µg/g/h	0.06	-	0.02	-	0.12	-	0.004	0.03	0.12	0.07	3
															

**B. Plants**															

13	Pine needle (PN) ^c^	ST/TD	MS	µg/L of HS	2.36	0.51	1.72	-	4.59	0.19	1.7	3.45	0.06	0.02	This study
				µg/g	0.59	0.13	0.43	-	1.15	0.05	0.42	0.86	0.02	0.004	
				µg/g/h	14.1	3.06	10.3	-	27.6	1.17	10.2	20.7	0.38	0.09	
14	Fresh Pine needle	LBC/DI	MS	µg/g	2398	523	258	758	170	-	-	204	-	-	4
15	Mixed grasses	BE/DI	FID	µg/m^2^/h	30.0	-	5.00	1	-	-	-	-	-	-	5 & 6
16	Bermuda grass	BE/DI	FID	µg/m^2^/h	2.00	-	-	6	-	-	-	-	-	-	5 & 6
17	pensacola grass	BE/DI	FID	µg/m^2^/h	7.00	-	2.00	-	-	-	-	-	-	-	5 & 6
18	Sawgrass	BE/DI	FID	µg/m^2^/h	62.0	-	10.0	-	-	-	-	-	-	-	5 & 6
19	*P. hortorum* leaf	MHS	MS	µg/m^2^	1.88	1.71	4.18	-	30.4	-	-	1.53	-	-	7
20	*P. hortorum* leaf	IMHS	MS	µg/m^2^	2.00	1.59	4.12	-	38.5	-	-	2.53	-	-	7
21	*P. hortorum* leaf	DHS	MS	µg/m^2^	2.35	1.82	4.65	-	41.6	-	-	3.00	-	-	7
														

**C. Fruit (beverages)**															

22	Orange juice (OJ) ^c^	ST/TD	MS	µg/L of HS	0.02	0.003	-	0.02	0.62	0.02	0.04	17.9	0.04	-	This study
				µg/g or µg/mL	0.004	0.001	-	0.005	0.15	0.005	0.01	4.47	0.01	-	
				µg/g/h	0.11	0.02	-	0.11	3.71	0.11	0.21	107	0.25	-	
23	Orange juice	SPME	FID	µg/L of HS	0.04	-	-	-	-	-	-	239	1.40	-	8
24	Orange wine	LLE	OL ^f^	µg/L	-	-	-	-	-	-	-	430	11.0	5.20	9

a Abbreviation: LVI-Large volume injection; HS-Headspace; DI-Direct injection; SPME-Solid phase microextraction; LLE-Liquid liquid extraction; LBC-Litter bag collection; BE-Bag enclosure; MHS-multiple headspace SPME IMHS-internal standard added multiple SPME; and DHS-dynamic headspace sampling;

b (1) [22]; (2) [23]; (3) [21]; (4) [24] (5 & 6) [25,27]; (7) [26]; (8) [28]; (9) [29];

c Sample mass placed on impinger: 1 g;

d Not detected;

e Emission flux = [(emission rate / sampling volume) × flow rate (0.1 L min^−1^)];

f Olfactometry.
