# Supplementary material for: Monoterpenes Released from Fruit, Plant, and Vegetable Systems
Source: Sensors (Basel). 2014 Sep 29;14(10):18286–301. doi: 10.3390/s141018286 (PMC4239866; doi:10.3390/s141018286)
Supplement: Supplementary file 1 [file sensors-14-18286-s001.pdf]

OPEN ACCESS
sensors
ISSN 1424-8220
www.mdpi.com/journal/sensors

Supplementary Information

Monoterpenes Released from Fruit, Plant, and Vegetable Systems. *Sensors* 2014, *14*, 18286-18301

Mohammad Asif Iqbal, Ki-Hyun Kim * and Jeong Hyeon Ahn

Atmospheric Environment Laboratory, Department of Civil and Environmental Engineering, Hanyang University, 222 Wangsimni-ro, Seoul 133-791, Korea;
E-Mails: iqbaldu88@gmail.com (M.A.I.); qqq112311@gmail.com (J.H.A.)

* Author to whom correspondence should be addressed; E-Mail: kkim61@hanyang.ac.kr or kkim61@nate.com; Tel.: +82-2-2220-2325; Fax: +82-2-2220-1945.

Table S1. Preparation procedures of liquid phase working standard (L-WS) of MTs used for ST/TD/GC analysis (by both MS and FID).

**A. Preparation of Liquid Phase Standard for 10 Target MTs Selected in This Study**													
	**Compounds**	**Methanol**	**α-PN**	**CMP**	**β-PN**	**3-CN**	**MRC**	**α-PD**	**α-TP**	***R*-LN**	**γ-TP**	***p*-CM**	**T**
**RGC** [a]	Purity (%)		99.0	95.0	98.5	98.5	95.0	95.0	95.0	99.0	98.5	99.0	99.5
**PS** [b]	Volume (μL)	8,790	1,000	1,050	1,000	1,000	1,160	1,000	1,000	1,000	1,000	1,000	1,000
	Concentration (ng/μL)		42,471	43,192	42,946	42,207	43,584	40,375	39,758	41,679	41,863	42,570	43,283
**1st WS** [c]	Volume (μL)	19,750	250	250	250	250	250	250	250	250	250	250	250
	Concentration (ng/μL)		531	540	537	528	545	505	497	521	523	532	541
**B. Preparation of L-WS for DI and ST- Based Calibration**													
**Order**	**Mixing Volume (μL)**		**Concentration** [d] **(ng/μL)**										
	**1st WS**	**Methanol**	**α-PN**	**CMP**	**β-PN**	**3-CN**	**MRC**	**α-PD**	**α-TP**	***R*-LN**	**γ-TP**	***p*-CM**	**T**
1	14	1,486	5	5	5	5	5	5	5	5	5	5	5
2	28	1,472	10	10	10	10	10	9	9	10	10	10	10
3	56	1,444	20	20	20	20	20	19	19	19	20	20	20
4	140	1,360	50	50	50	49	51	47	46	49	49	50	50
5	280	1,220	99	101	100	98	102	94	93	97	98	99	101
6	850	650	301	306	304	299	309	286	282	295	297	302	307

[a] Reagent grade chemicals; [b] PS: Dilution of pure chemicals (reagent grade chemical) to make 20 mL solution; [c] 1st L-WS: Dilution of PS to make in 20 mL solution; [d] Injection/loading volume on ST: 1 μL.

Table S2. Instrumental setup and operational conditions for the gas chromatographic analysis of MTs.

**A. Basic Information of Sorbent Tubes (STs) Prepared for the Absorption of MTs**					
Sorbent material- Tenax TA + Carbopack B + Carbopack X			Short name	TBX	
Tube material	Quartz		Amount of each sorbent	50 mg (all in quartz)	
Sorbents separated by	Quartz wool		Purge/sweeping gas	N_2 (99.999 %)	
**B. GC/MS System: QP2010, Shimadzu, Japan**			**(b)** Detector (MS)		
**(a)** Oven setting			Ionization mode:	EI (70 eV)	
Initial temp.:	80	°C	Ion source temp.:	200	°C
Ramping rate:	1	°C min^{-1}	TIC scan range:	35~250	m/z
Final temp:	90	°C	Threshold:	100	
Initial hold time	0	min	**(c)** Column (CP-WAX column, Varian, USA)		
Final hold time:	5	min	Length (*l*)	60	m
Total run time	15	min	Internal diameter (*id*)	0.25	mm
Carrier gas:	He	99.999%	Film thickness:	0.25	µm
**C. GC/FID: Varian GC, Agilent Tech, USA; Column: CP-WAX (*l*: 30 m, *id*: 0.25 mm, and film thickness: 0.25 µm)**					
**(a)** Oven setting			**(b)** Detector setting		
Initial temp	75	°C	Detector temp.	250	°C
Initial rate	2	°C·min^{-1}	H_2 and N_2 flow	30	mL·min^{-1}
Initial Hold	1	min	N_2 flow	30	mL·min^{-1}
Final temp	90	°C	Air flow	300	mL·min^{-1}
Final Hold	1.5	min	Carrier gas N_2	1	mL·min^{-1}
Total time	10	min			
**D. Thermal Desorber (TD): UNITY, Markes International, Ltd., UK [a]**					
Cold trap:	Carbopack C + Carbopack B				
split ratio:	1:5		trap low:	−10	°C
split flow:	20	mL·min^{-1}	trap high:	300	°C
trap hold time:	5	min	flow path temp.	120	°C
desorption time:	10	min	Desorption temp.	300	°C

[a] TD setup was identical for both GC/FID and GC/MS analysis.

Table S3. Basic quality assurance (QA) parameters for target MTs analyzed by both FID and MS.

Order	Compounds	MDL [a]				RSE [b] (%)	
		TD/GC/MS		TD/GC/FID		MS	FID
		Mass (ng)	Concentration (ppb) [c]	Mass (ng)	Concentration (ppb)		
1	α-PN	0.24	0.09	0.38	0.14	0.50	1.17
2	CMP	0.23	0.08	0.89	0.32	1.06	0.5
3	β-PN	0.38	0.14	0.72	0.26	3.76	2.04
4	3-CN	0.40	0.15	0.53	0.19	0.63	4.04
5	MRC	0.24	0.09	0.54	0.19	1.67	2.17
6	α-PD	0.25	0.09	0.72	0.26	1.66	1.10
7	α-TP	0.41	0.15	0.64	0.23	2.23	1.79
8	R-LN	0.50	0.18	0.62	0.22	0.83	1.43
9	γ-TP	0.34	0.12	0.58	0.21	1.02	2.48
10	p-CM	0.29	0.11	0.6	0.22	0.85	0.66
11	T	0.56	0.30	0.43	0.23	2.12	1.24
Average MDL & RSE values for all MTs		0.33	0.12	0.62	0.22	1.42	1.74

[a] MDL values were obtained by conducting seven replicate analyses of 1 μL liquid standard; (5 ng·μL^{-1}) loaded on to ST with the supply of 0.5 L N_2 purging gas; [b] Derived by triplicate analyses of 1 μL of liquid standard (5 ng·μL^{-1}) loaded on to ST; [c] DL values for gas standards were calculated in ppb unit assuming the total sample volume of 0.5 L.

© 2014 by the authors; licensee MDPI, Basel, Switzerland. This article is an open access article distributed under the terms and conditions of the Creative Commons Attribution license (http://creativecommons.org/licenses/by/4.0/).
